# Insight into gastrointestinal heterotopic pancreas: imaging evaluation and differential diagnosis

**DOI:** 10.1186/s13244-021-01089-0

**Published:** 2021-10-21

**Authors:** Cai-Wei Yang, Feng Che, Xi-Jiao Liu, Yuan Yin, Bo Zhang, Bin Song

**Affiliations:** 1 West China School of Medicine, Sichuan University, Chengdu, 610041 China; 2 Department of Radiology, West China Hospital, Sichuan University, Chengdu, 610041 Sichuan Province China; 3 Department of Gastrointestinal Surgery, West China Hospital, Sichuan University, Chengdu, 610041 Sichuan Province China

**Keywords:** Gastrointestinal heterotopic pancreas, Computed tomography, Imaging features, Gastrointestinal subepithelial tumors

## Abstract

Heterotopic pancreas (HP) is an uncommon congenital abnormality in the developmental process of the pancreas, with gastrointestinal heterotopic pancreas (GHP) being the most common HP. The clinical manifestations of GHP may have variable patterns of presentation, dictated by both the anatomic location and the functional ability of the lesion. The most common imaging modality in detecting GHP is computed tomography (CT), while gastrointestinal barium fluoroscopy, endoscopic ultrasonography, and magnetic resonance imaging (MRI) are also applied. The density and enhancement patterns of GHP are consistent with histological classifications. GHP with a predominantly acinar tissue component manifests homogeneous and marked enhancement on CT images, whereas a predominantly ductal GHP presents heterogeneous and mild enhancement. On MRI, the appearance and signal intensity of GHP were paralleled to the normal pancreas on all sequences and were characterized by T1-weighted high signal and early marked enhancement. This article provides a comprehensive review of the histopathology, clinical manifestations, imaging features of various modalities, and differential diagnosis of GHP. It is hoped that this review will improve clinicians’ knowledge of GHP and aid in accurate preoperative diagnosis, thereby reducing the misdiagnosis rate.

## Key points

- Gastrointestinal heterotopic pancreas is an uncommon congenital abnormality in the developmental process of the pancreas.
- The clinical manifestations of gastrointestinal heterotopic pancreas may have variable presentations decided by both the anatomic location and the functional ability of the lesion.
- Some distinct imaging features of various modalities may support the diagnosis of gastrointestinal heterotopic pancreas.
- Gastrointestinal heterotopic pancreas and other gastrointestinal subepithelial tumors could be differentiated by non-invasive imaging.

## Introduction

Heterotopic pancreas (HP) is a kind of congenital abnormality occurring during the developmental process of the normal pancreas, also known as ectopic pancreas, aberrant or accessory pancreas, pancreatic choristoma, or adenomyoma [1–4]. The true prevalence of HP is difficult to assess because most patients have no clinically significant symptoms. HP is found intraoperatively in approximate 0.2% of unrelated upper abdominal surgeries and 0.9% of gastrostomies [5, 6]. Autopsy results reveal the incidence of HP to be 0.5–13.7% approximately. The disease is most often seen in males, and the incidence peaks in the 4th, 5th, and 6th decades of life [6, 7]. HP lesions can arise in tissues throughout the upper gastrointestinal tract system, with surgical and autopsy data reporting a frequency within the stomach of 25–52%, 27–36% in the duodenum, and 15–17% in the jejunum, respectively [8]. Less common sites of HP include the ileum, esophagus, and Meckel's diverticulum, but also occasionally in the mesentery, hepatobiliary system, spleen, mediastinum, lung, and umbilical foramen [5]. This leads to further complexity in accurate diagnosis and treatment of HP as it is commonly confused with other disease processes.

**Fig. 1:**
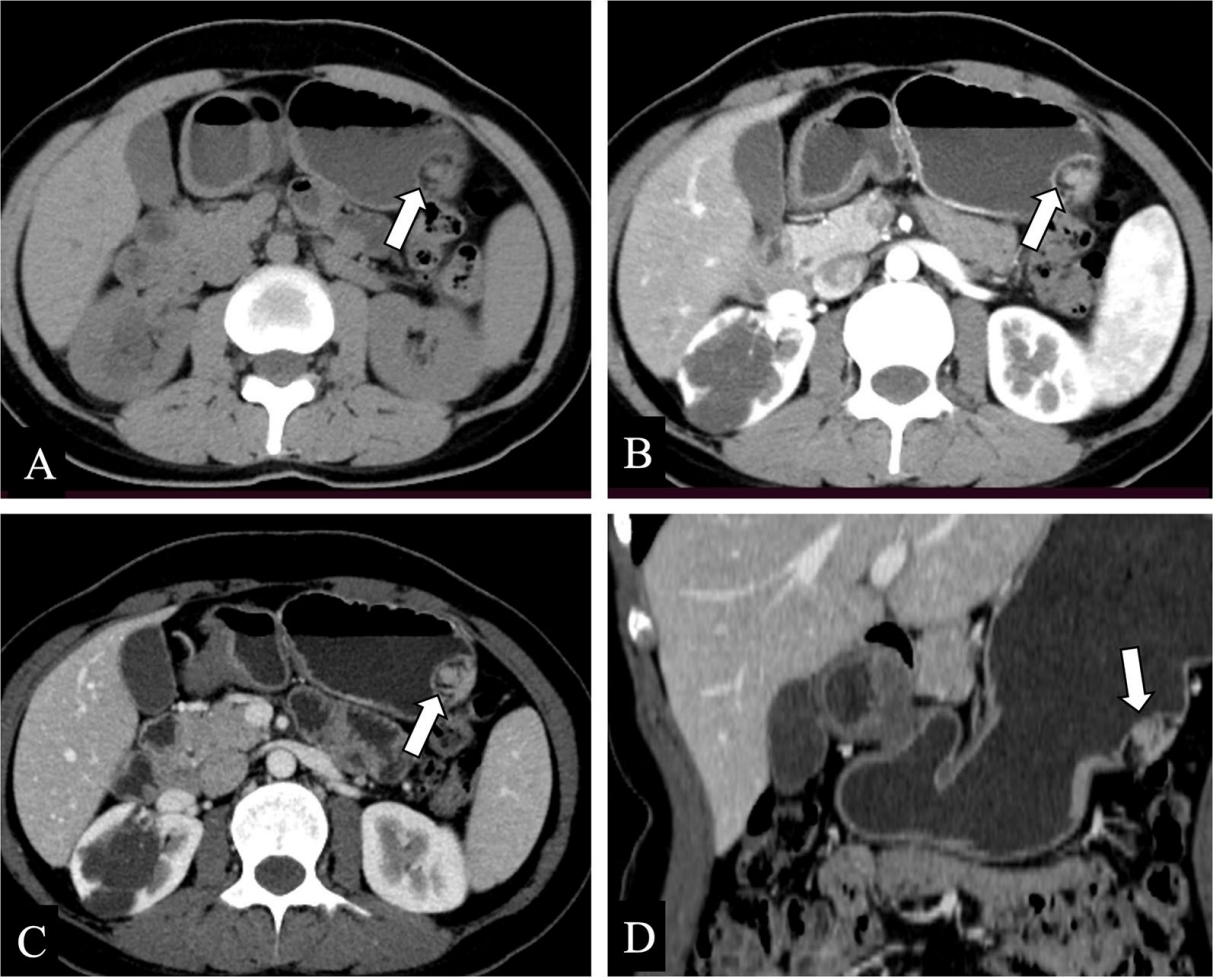
**a**–**d** Axial plain/arterial/venous phases CT images of a 33-year-old female showed a 2.3 × 1.7 cm, round, and micro-lobulated subepithelial lesion (white arrows), indicating an endoluminal growth pattern of a heterotopic pancreas in the gastric body (**a**–**c**). **d** Coronal venous phase CT image

Gastrointestinal heterotopic pancreas (GHP) is essentially analogous to the normal pancreas in terms of gross and histological specimen. GHP can be classified as a subepithelial lesion, which is defined as a mass covered by normal mucosa [9]. GHP appears as a solid intramural mass with a micro-lobulated border that is not clearly demarcated from the surrounding tissues [10]. GHP is mostly solitary (80%), and its diameter is mostly less than 3 cm, but the size also varies from 0.2 cm to 5.0 cm (Fig. 1) [10, 11].

### Clinical manifestations

**Fig. 2:**
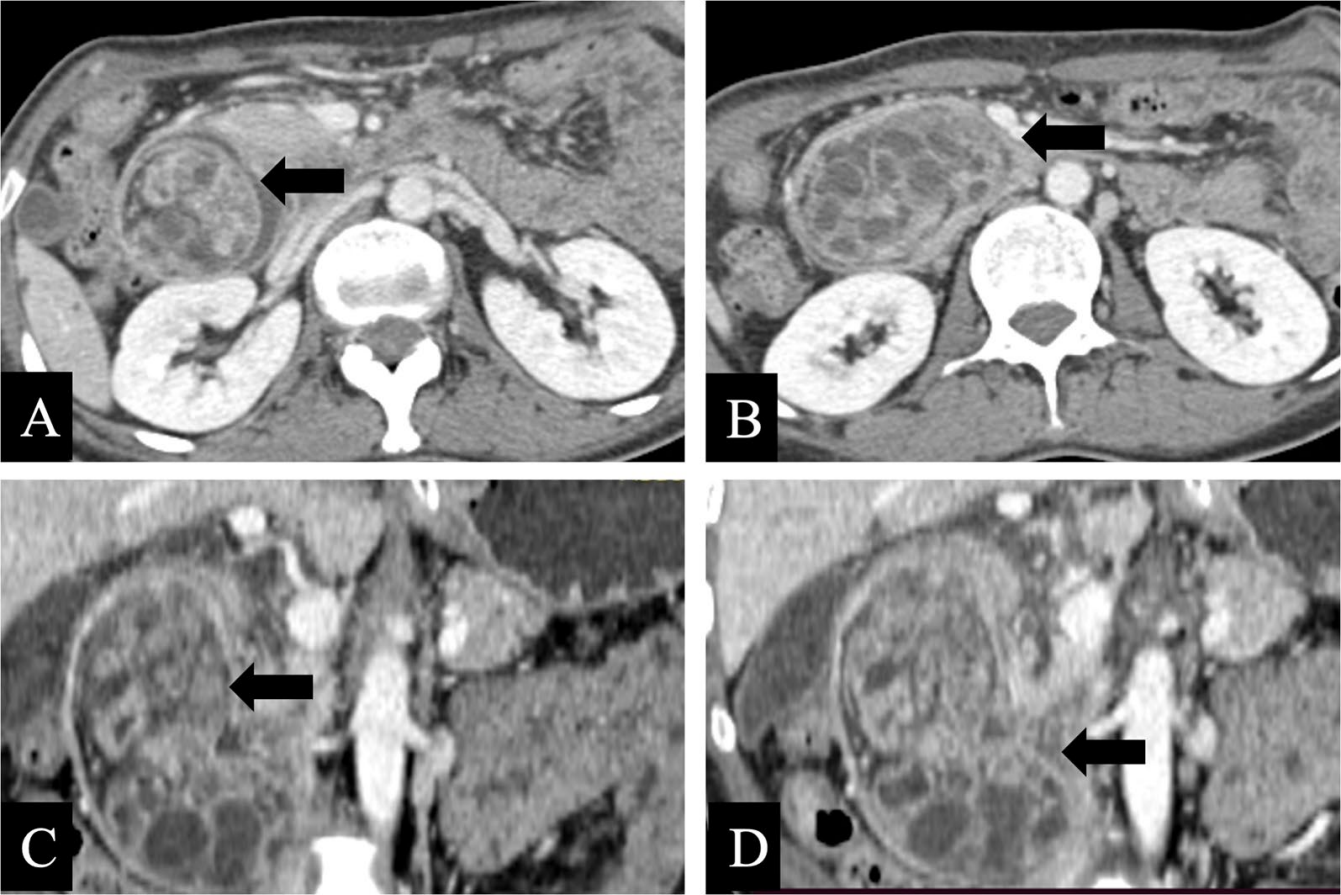
**a–d** A 46-year-old female presented with abdominal pain and anorexia over the course of one year with a weight loss. Contrast-enhanced axial CT images presented a 5.8 × 4.9 cm, endoluminal, and ill-defined mass (black arrows) in the duodenum accompanied by subsequent gastric outlet obstruction with surrounding inflammation and duodenal intussusception, and the patient was noted to have mild elevation in her amylase and lipase. The patient was subsequently taken to the operating room for antrectomy and Billroth II reconstruction. Her pathology demonstrated a heterotopic pancreas in the duodenum

**Fig. 3:**
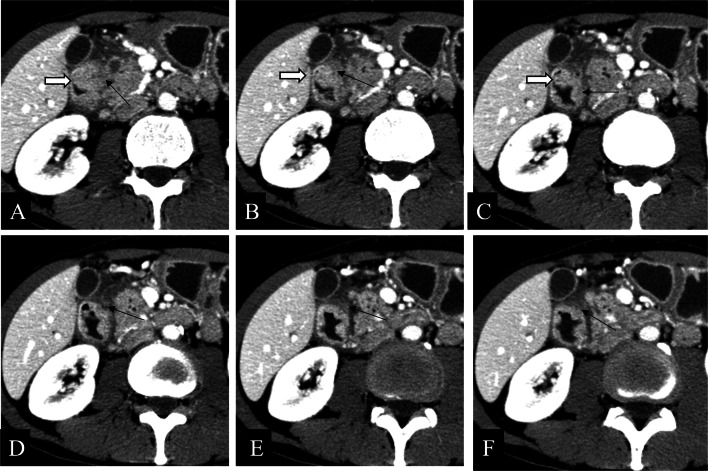
Groove pancreatitis in a 44-year-old male. **a–f** Axial contrast-enhanced CT images showed a sheetlike hypoattenuating mass (black arrows) in the pancreaticoduodenal groove and separated from the pancreatic head and the duodenum, with thickening of adjacent medial wall of the duodenum. **a–c** The final pathology demonstrated a 2.7 cm sized heterotopic pancreas in the duodenum (white arrows)

Patients with GHP are often asymptomatic, but some patients may illicit significant clinical manifestations. Characterization of GHP symptomatic lesions is slightly difficult due to the relative infrequency of this diagnosis and the variability and non-specificity in presentation, often leading to misidentification and suboptimal management in many cases. GHP is susceptible to the same pathological conditions as the normal pancreas, so its complications are like those of the normal pancreas, such as pancreatitis, pancreatic pseudocysts, even malignancy. In severe cases, it may cause acute abdomen such as gastrointestinal hemorrhage (hematemesis or melena), or even intestinal obstruction and intussusception (Fig. 2). Some complications may be misdiagnosed as malignant neoplasm. Groove pancreatitis is considered as an associated disease related to GHP, with its most important etiology to be cystic degeneration and fibrosis of the heterotopic pancreatic tissue embedded in the pancreaticoduodenal groove (Fig. 3).

Esophageal lesions often bring about progressive dysphagia or epigastric pain, occasionally leading to gastroesophageal reflux and hiccup, and rarely hematemesis [12]. Secondary inflammation of the submucosal gastric lesions may lead to non-atrophic gastritis, but mucosal ulcers are rarely formed [13, 14]. Pyloric lesions may cause gastrointestinal obstruction. Jejunal lesions may act as a leading point for intestinal obstruction. Hepatopancreatic ampullary lesions give rise to biliary obstruction [5]. GHP in the hepatobiliary system tract is rare and rarely symptomatic [15], but it occasionally causes bile duct dilatation, bile duct obstruction, gallbladder effusion, cholecystitis, and even gallbladder perforation, which may be related to its heterotopic location. And its complications are not significantly distinct from other hepatobiliary systematic lesions [16].

In addition to the anatomic location, histological location within the layers of the visceral wall may also influence symptomatic presentation. GHP most commonly occurs between the submucosa and lamina propria but can be found in all layers of the visceral wall [14]. Submucosal lesions may be more likely to cause ulceration in local gastritis or duodenitis, while transmural lesions involving all layers of the bowel wall can lead to chronic inflammation and ultimately stricture or perforation [17].

Although location can often explain symptomatic presentation, it does not fully explain why some lesions are symptomatic and others found in similar locations are not. The remaining factor can be explained by the function of the lesion and the ability to perform the normal exocrine and endocrine functions of the pancreas. A histopathological evaluation by Heinrich in 1909 showed differences in the composition of these lesions which were later revised by Fuentes in 1973 [18, 19]. These classification systems classify GHP lesions according to the presence of all cellular components of functional pancreas tissues (Type I in both categories) and the presence of ducts, acinar tissue, or islet cells (Fuentes classification types II, III, and IV, respectively) [18, 19]. The presence or absence of histological elements may affect the function of the lesion and result in its ability (or inability) to produce symptoms. It has been proposed that lesions with functional exocrine potential may produce local chemical irritation of surrounding tissues, while lesions without appropriate duct drainage may lead to pancreatitis and the formation of pseudocysts within the lesion [20, 21].

### Imaging evaluation of various imaging modalities

Accurate identification of GHP during diagnostic tests is important in determining the appropriate management. Unfortunately, many patients are misdiagnosed at the time of surgery or are thought to have other pathological changes. This often influences surgical decisions making and can lead to a more extensive resection than would otherwise be required. This was evident in the study by Zhang et al.*,* which reported that over 54% of patients with GHP were misdiagnosed preoperatively [22]. Many lesions were considered malignant and underwent extensive resections. An accurate diagnosis of GHP, regardless of manifesting symptoms, may alter management and influence surgical decision. If the presence of GHP appears likely, appropriate imaging and diagnostic studies must be carefully evaluated. Lesions of adequate size can be identified by non-invasive imaging examination. Gastric HP is most located in the greater curvature side of the gastric antrum, within 6 cm to the pylorus [14]. Duodenal lesions are often posited in the descending duodenum, and jejunal lesions are most near the Treitz ligament [23]. Esophageal lesions are mostly placed in the distal third part of the esophagus [12].

In the past, the common imaging modality for GHP was gastrointestinal barium fluoroscopy. With the development of imaging technology, endoscopic ultrasonography (EUS), contrast-enhanced computed tomography (CT), and magnetic resonance imaging (MRI) are increasingly used for the detection and follow-up of GHP. Currently, the most applied imaging modality for GHP description is contrast-enhanced CT.

### Upper gastrointestinal barium fluoroscopy

**Fig. 4:**
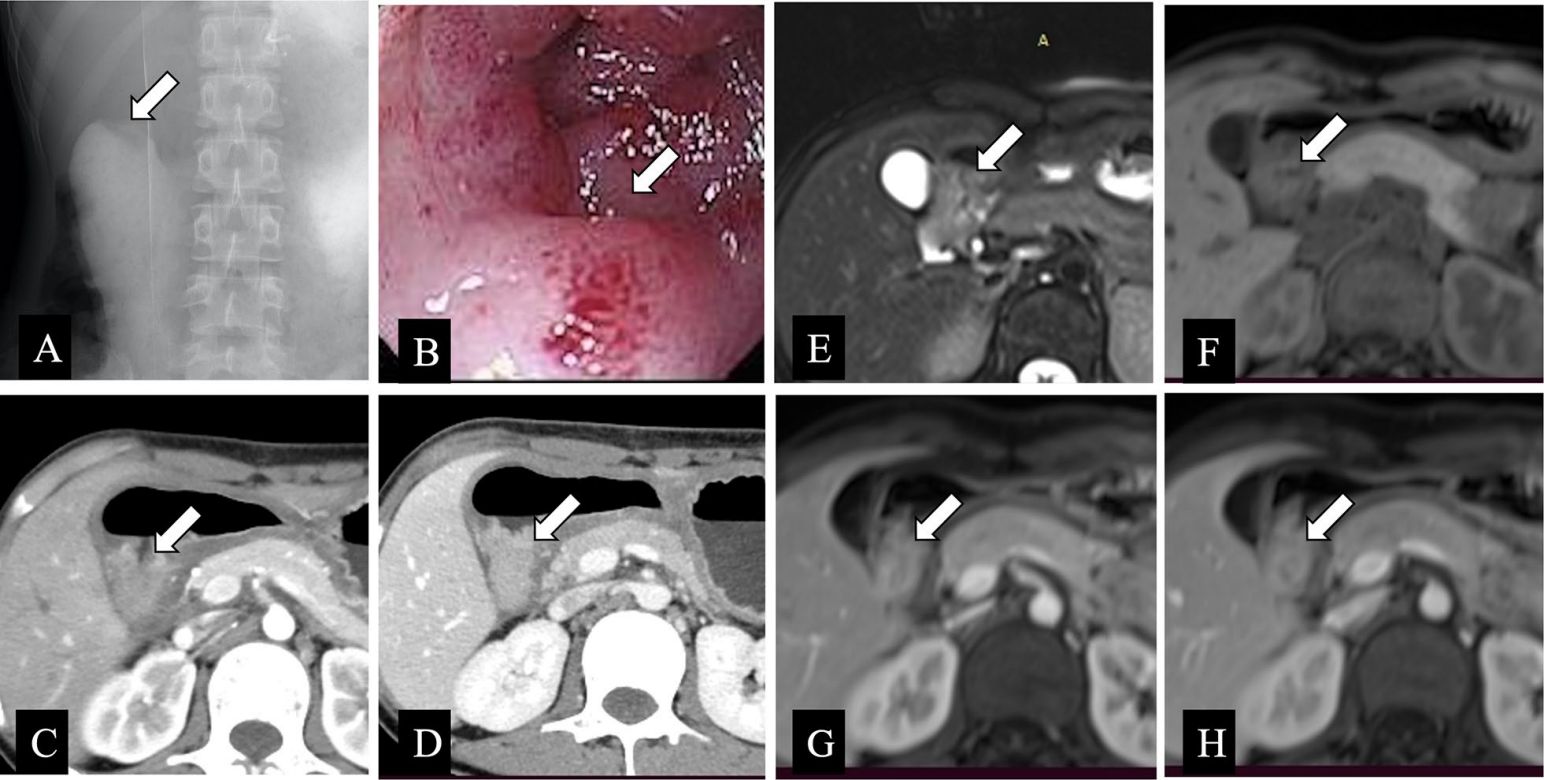
A 15-year-old female presented with abdominal pain and distension underwent various imaging examinations. **a** Single spot image of the upper gastrointestinal tract on a barium fluoroscopic study demonstrated an intraluminal filling defect within the duodenum with a central indentation (white arrow) consistent with heterotopic pancreas. **b** Endoscopy showed a subepithelial lesion (white arrow) along the first portion of the duodenum with surrounding duodenitis. **c–d** Contrast-enhanced axial CT images revealed a hyper-enhanced lesion (white arrows) with ill-defined margin and thickening of the surrounding duodenal wall. **e–h** Non-contrast axial T2-weighted (**e**), pre-contrast T1-weighted (**f**), post-contrast arterial T1-weighted (**g**), and post-contrast venous T1-weighted images (**h**) showed a lesion within the first portion of the duodenum (white arrows) demonstrating T2-weighted slightly hyperintense signal, pre-contrast T1-weighted slightly hypointense signal, and post-contrast T1-weighted isointense like those of the normal pancreas

On barium examination, GHP has a typical appearance of an intramural tumor with a broad base and a smooth surface [10, 24]. An ulcer-like barium spot in the center of the lesion, which may be the rudimentary ductal drainage system of GHP, known as “umbilication” or “central umbilical sign,” is the characteristic imaging feature to differentiate GHP from other intramural gastric submucosal tumors. In one study [24], one fifth of GHP were detected typical “central umbilical sign,” and a large barium umbilication that resembled an ulcer or an ulcerative tumor was rare (Fig. 4) [24].

### Endoscopic ultrasonography

**Fig. 5:**
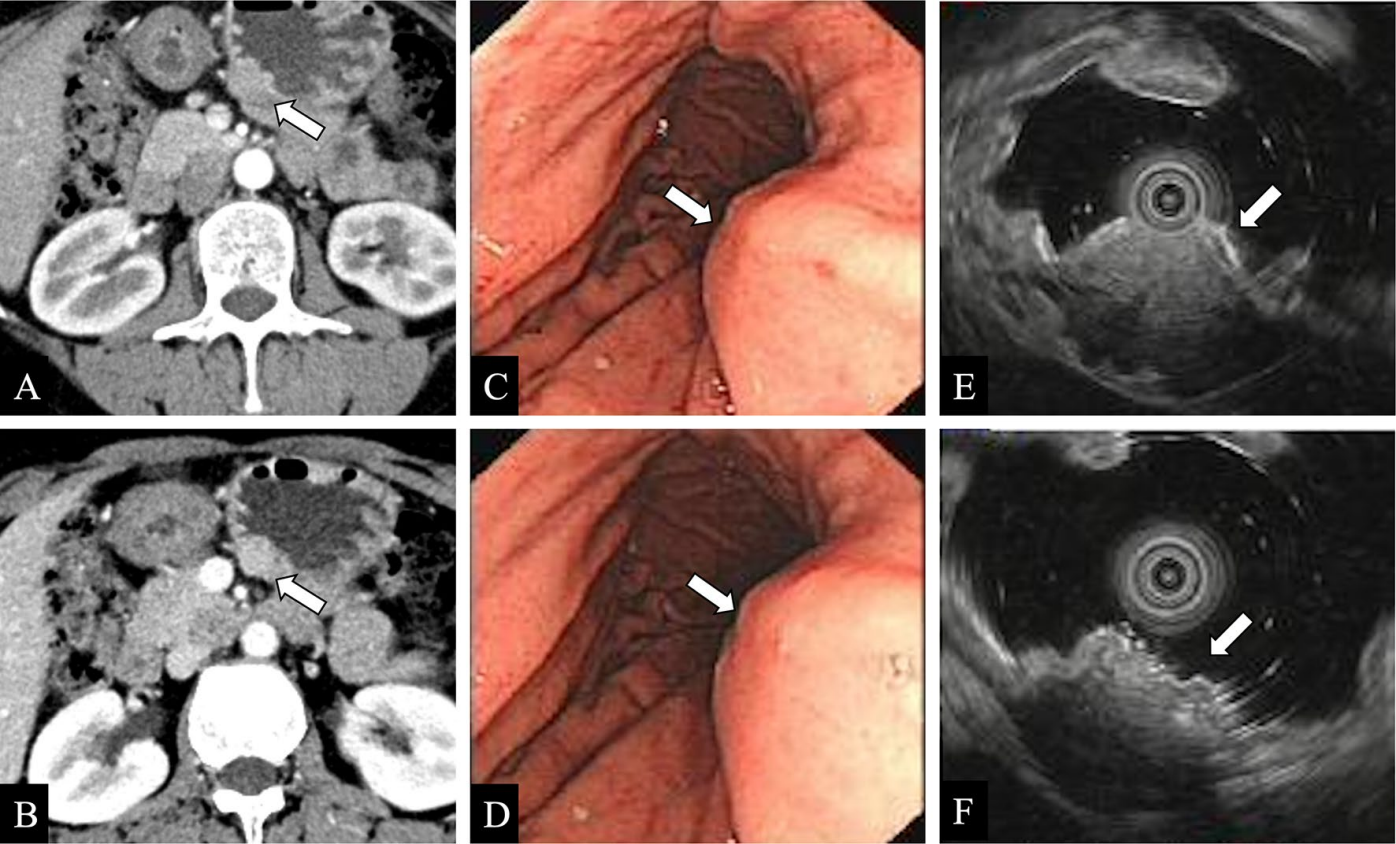
Images demonstrating the gastric body with a submucosa heterotopic pancreas (white arrows) identified within the wall of the stomach. **a, b** Contrast-enhanced axial CT images in arterial and venous phases. **c, d** Upper endoscopy images. **e, f** Endoscopic ultrasonography images

Endoscopically, GHP shows as an endoluminal submucosal mass [6, 25]. Studies have shown that “central umbilical sign” is more easily detected in endoscopy than in conventional imaging [6]. Superficial endoscopic biopsies are often unable to diagnose GHP because of its subepithelial location and can also be difficult in the setting of ulceration, cystic degeneration, and in lesions that are located within the outer wall of the viscera [13, 26]. GHP most often presenting as a solid submucosal mass is hypoechoic relative to the mucosa and isoechoic relative to the mucosal muscle layer of gastrointestinal wall on EUS (Fig. 5). Further, EUS may help facilitate accurate targeting, delineate the contour and location of the lesion in the intestinal wall, and perform a fine-needle aspiration biopsy, which compensates for the deficiency of superficial endoscopic biopsy [5, 26–29].

### Contrast-enhanced computed tomography

**Fig. 6:**
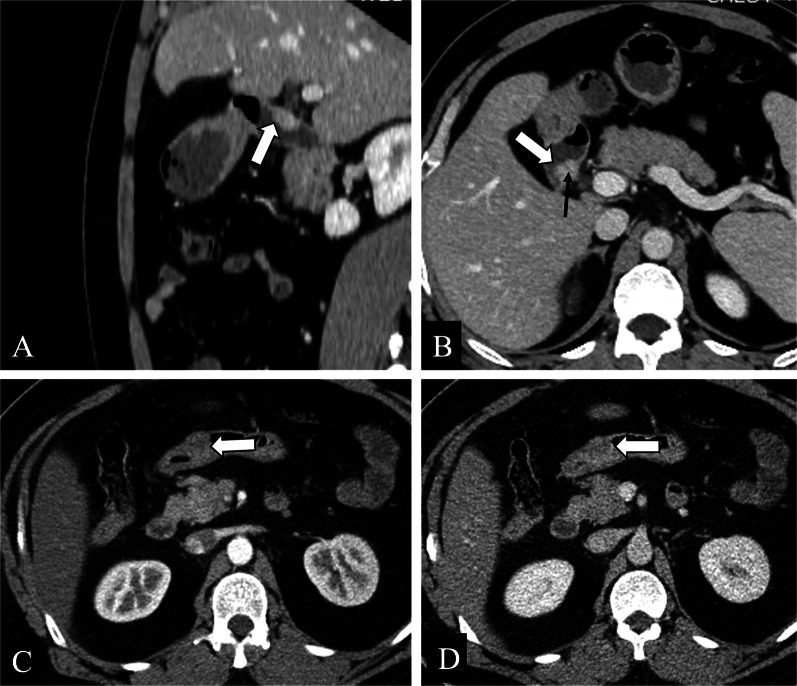
**a, b** Coronal and axial enhanced CT images of a heterotopic pancreas in the duodenum of a 53-year-old male showed a 1.3 cm × 0.9 cm, endoluminal lesion (white arrows) with a lobulated margin, presenting round contour on axial image while flat pattern on coronal CT image. Small duct-like structure (black arrow) was seen within the lesion, which measured with a long diameter to short diameter > 1.4 on coronal CT image. **c, d** Axial enhanced CT images of a heterotopic pancreas in the gastric antrum of a 58-year-old male showed a 1.2 cm × 0.8 cm, oval, ill-defined, and endoluminal lesion (white arrows), with a long diameter to short diameter > 1.4

**Fig. 7:**
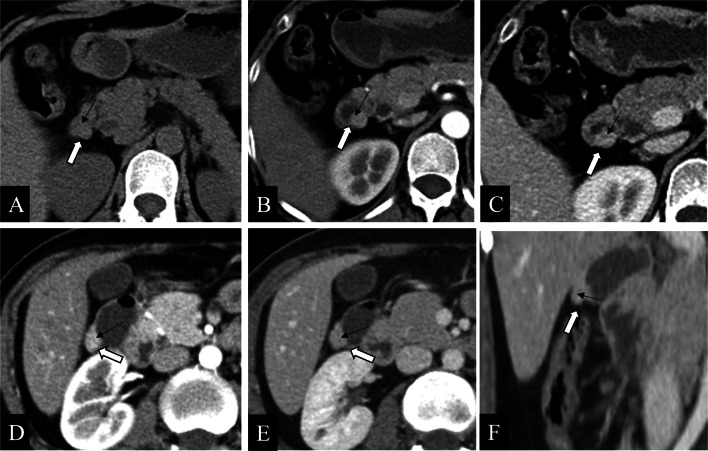
**a–c** Axial plain (**a**) /arterial (**b**) /venous (**c**) phases CT images of a heterotopic pancreas in the duodenum of a 57-year-old female showed a 1.2 cm, round, well-defined, and mixed-growing lesion (white arrows). Small duct-like structure (black arrows) was vaguely seen within the lesion in each phase, presenting slightly hypo density compared to the surroundings. **d–f** Axial arterial (**d**) /Venous (**e**) /coronal venous (**f**) phases CT images of a heterotopic pancreas in the duodenum of a 58-year-old male showed a 1.3 cm × 0.9 cm, flat lesion (white arrows) with a lobulated contour and an exophytic growth pattern. Small duct-like structure (black arrows) was seen within the lesion in each phase

GHP often presents as an intramural oval mass with indistinct margins on CT coronal images [23, 30]. GHP is usually small in diameter (often less than 3 cm) and tends to be an intraluminal growth pattern [6, 14, 31, 32]. The CT attenuation, enhancement heterogeneity, and enhancement degree of GHP correlate with its pathological histological compositions [5, 9, 10, 14, 23, 33]. It has been shown that the type with similar or stronger enhancement than the normal pancreas is dominated by acinar tissues, whereas the less enhancement type than the normal pancreas is composed mostly of ductal structures and hyperplastic muscular layers. Similarly, homogeneous enhancement pattern has a microscopic mainly consist of acinar component, whereas heterogeneity enhancement pattern gives priority to ductal component [6, 10, 14, 23]. The lesion margin seen on CT images is indistinct and slightly micro-foliated, consistent with the histopathology in which the alveoli is composed of a lobular morphology [14, 28]. Moreover, “a duct-like structure” imaging feature of GHP can be found as a thin hypodense strip of shadow in all phases of contrast-enhanced CT images, which varied in size from about 1 to 5 mm in width and 5 to 10 mm in depth [34]. It can be observed microscopically that the duct-like tissue of GHP communicates with the gastrointestinal lumen, but this may be difficult to observe on axial CT images [14, 23]. Rather, a study indicated that coronal and sagittal observation based on three-dimensional reconstruction may improve the characteristic sign detection of “a duct-like structure” (Fig. 6) [31]. GHP in the jejunum shares many same imaging features as those in the stomach and duodenum, but there are a few disparities: (a) while “central umbilical sign” and hyperenhancement of underlying mucosa of adjacent gastrointestinal wall can occasionally be observed in the gastric and duodenal lesion, both are rare in the jejunal lesion [23], (b) GHP in the stomach or duodenum manifest endoluminal growth pattern predominantly, whereas no dominant growth pattern was found in jejunal lesion: endoluminal, exophytic, and mixed growth patterns were all distributed [23], (c) “a duct-like structure” sign in the jejunal lesion is relatively more difficult to identify than that in the gastric lesion [10, 23], and a study found that only three cases (3/16, 19%) of jejunal lesions were observed “a duct-like structure” signs on CT images [23]. Gastric HP is often endoluminal growing, mostly located in the antrum, with an oval or flat shape, and the ratio of long diameter (LD) to short diameter (SD) is likely greater than 1.3 or 1.4 (Fig. 7) [14, 32]. Gastric HP grows internally into the intestinal lumen without distorting its external contour, contrary to the pattern growing exophytic from the intestinal wall without bulging into the lumen. In contrast, there is also a proportion of round HP lesion in the duodenum or jejunum, which has been suggested to result from the tension difference existing between the intestinal wall and the gastric wall [6, 23].

### Magnetic resonance imaging

**Fig. 8:**
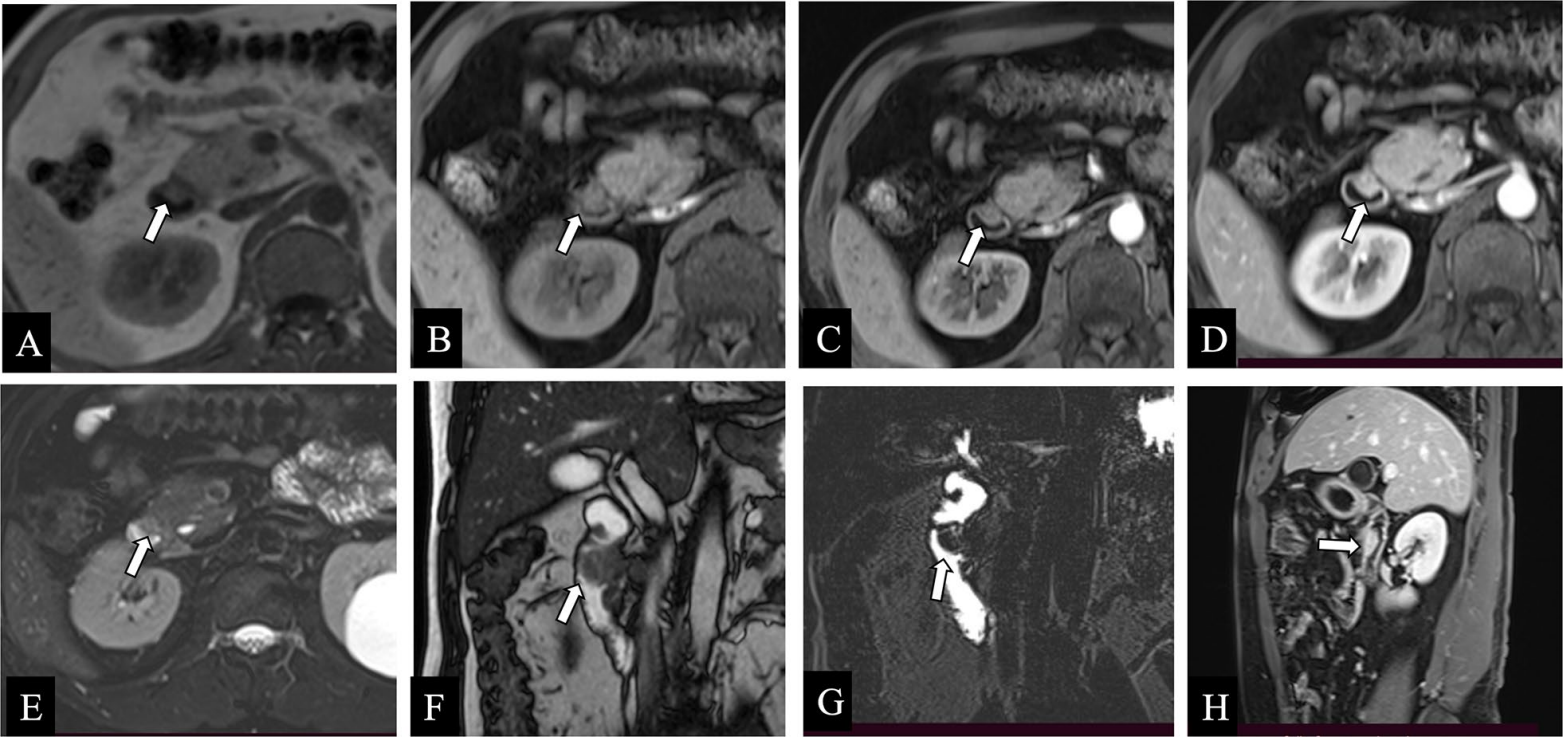
**a–h** Non-contrast axial T1-weighted (**a**), pre-contrast axial T1-weighted (**b**), post-contrast axial T1-weighted (**c, d**), and post-contrast coronal T1-weighted (**h**) images showed a lesion within the duodenum (white arrows) demonstrating T1-weighted hyperintense or isointense signal like those of the normal pancreas. This tissue showed similar imaging characteristics of the normal pancreas on axial T2-weighted (**e**) and coronal true fast image with steady-state precession (True-FISP) (**f**) images (white arrows). Magnetic resonance cholangiopancreatography image (**g**) showed a nodular filling defect in the duodenum

The appearance and signal intensity of GHP resembles that of the normal pancreas in all MRI sequences [5, 35]. GHP shows characteristic discriminatory high signal on T1-weighted images [5]. GHP and the normal pancreas often appear similar signal intensity and paralleled enhancement degree in associated MRI sequences [35], with the lesion exhibiting marked enhancement in the late arterial phase. But some GHP may display more pronounced enhancement than the normal pancreas. Magnetic resonance cholangiopancreatography (MRCP) plays an important auxiliary role in identifying the rudimentary ductal system. The dilated duct of GHP, also known as the “heterotopic duct sign,” is more easily manifested on T2-weighted images and MRCP images, a specific sign that helps to distinguish GHP from other submucosal lesions (Fig. 8) [5, 36].

### Differential diagnostic performance between GHP and other gastrointestinal subepithelial tumors

GHP is still prone to misdiagnosis in clinical practice. Identifying the imaging features of GHP can help to distinguish it from tumors and thus avoid unnecessary surgery. The main differential diagnostic tumors of GHP are other gastrointestinal subepithelial tumors, including gastrointestinal stromal tumor (GIST), gastroduodenal glomus tumor (GGT), schwannomas, leiomyomas, and so on. The differential diagnosis of the complications of GHP pancreatitis includes groove pancreatitis, myoadenomatosis, pancreatic hamartoma of the duodenum, pancreatic adenocarcinoma involving the biliary tree, and so on [37–39]. The non-invasive imaging assessment literatures centered on contrast-enhanced CT, EUS, and MRI.

### Contrast-enhanced computed tomography

Contrast-enhanced CT is the most applied imaging modality for differentiating GHP from other subepithelial tumors. Kim et al. [14] initially proposed a research using sensitivity, specificity, odds ratio, and combination of variables to investigate the role of contrast-enhanced CT for differentiating GHP from GIST and gastrointestinal leiomyoma with qualitative and quantitative imaging features. It firstly evinced a morphological growing method with a LD to SD ratio to assess GHP and other subepithelial tumors. When significant identifying features were used in combination, the values of sensitivity and specificity were improved somehow. Then, a similar study applied above features and methods to evaluate GGT from other subepithelial lesions including GHP and GIST [40]. Although it only focused on GGT, its quantitative parameters introduced an approach of quantification and precision of the lesion CT attenuation value: using the lesion to aorta ratio in each phase [40].

Nevertheless, previous studies didn’t make usage of receiver operating characteristic analysis to determine the diagnostic performance of contrast-enhanced CT. Later, studies filled the gap by addressing the shortcoming, though lack of pathologic to radiologic correlation [31, 32, 41]. Compared to Kim et al.’s study, Yang et al. [31] not only individualized CT attenuation by using the lesion to aorta ratio method, but also combined significant variables in univariate analysis and achieved higher area under the curve values. Liu et al. [41] and Li et al. [32] both focused on the differentiation of gastric HP from gastric stromal tumors (GST); they have some differences and emphases. Liu et al. [41] collected all GST in their study with a diameter limitation of ≥ 1 cm and calculated the CT attenuation disparities by using the enhanced CT attenuation value minus that of unenhanced phase while Li et al. [32] limited the lesion diameter of < 3 cm, utilized stratified random sampling method to rate same sample sizes of gastric HP and GST, and employed the group validation method to prove the drawn conclusion of the primary cohort. Further, the lesion-to-pancreas ratio in venous phase was operated. It manifested that significant variables in the primary cohort were also significant in the validation cohort, which emphasized and evidenced the authenticity and reliability of research results.

### Endoscopic ultrasonography

At present, studies of EUS bring into play a role in describing and figuring the endosonographic features of GHP [28, 42]. Few studies have concentrated on distinguishing GHP from other subepithelial tumors. An endosonographic study evaluated the morphological and echoic features of gastric HP, GIST, and other mesenchymal tumors and came to significant conclusions [42]. Park et al. [28] compared the efficacy between CT combined EUS and CT only in differentiating endoscopically suspected gastroduodenal HP from GIST and other subepithelial lesions. With its five-point scale scoring evaluation system, they revealed that CT and EUS were both useful, and CT combined EUS signified a superior diagnostic accuracy than CT only significantly [28]. They pointed out that each imaging technique has its dominances and drawbacks, which might germinate conceptions on the combination of various imaging features of a single modality and combination of various imaging modalities in evaluation.

### Magnetic resonance imaging

MRI has a superior ability in identifying GHP based on its multi-parameters imaging and multiple sequences, especially on MRCP and T2-weighted images. The only study assessed MRI with diffusion-weighted imaging findings of GHP and other gastrointestinal submucosal tumors, suggesting that qualitative and quantitative parameters could be helpful in differentiation diagnosis [43]. Preoperative MRI is supposed to be more widely applied in submucosal tumors for non-invasive detection and differentiation.

### Main differentiation: features of other gastrointestinal subepithelial tumors

#### Gastrointestinal stromal tumor

**Fig. 9:**
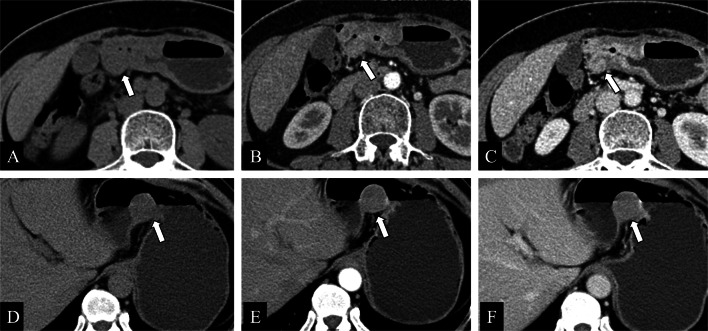
**a–c** Heterotopic pancreas in a 61-year-old female. Axial contrast-enhanced CT images obtained during the plain (**a**) /arterial (**b**) /venous (**c**) phases showed a heterogeneous enhanced flat mass (white arrows) in the gastric antrum. The mass had an endoluminal growth pattern, which is more common with heterotopic pancreatic lesion in the stomach. **d–f** Gastrointestinal stromal tumor in a 41-year-old male. Axial contrast-enhanced CT images obtained during the plain (**d**) /arterial (**e**) /venous (**f**) phases also showed a homogeneous enhancing round mass (white arrows) in the gastric antrum with the same endoluminal growth pattern

GIST is the most common submucosal tumor that originates from interstitial Cajal cells in the muscular layer of the gastrointestinal wall and can occur anywhere along the digestive tract. The most common location of GST is the fundus or body [14]. Most of GIST show exophytic growth or mixed growth, with a mixed growth pattern including both endoluminal and exophytic parts: in a dumbbell shape, a growth pattern that is rare in GHP (Fig. 9) [23].

The typical appearance of primary GIST is a submucosal mass with irregular contour and heterogeneous enhancement, which may be accompanied by mucosal ulceration, necrosis, cystic degeneration, hemorrhage, calcification, and presentation of enlarged draining vessels [44]. Nearly half of GIST patients are found with metastases, and the liver and peritoneum are organs most likely to be involved [45]. GIST smaller than 2 cm is often endoluminal growing with well-defined border, presenting homogeneous hypodense attenuation, so it might be challenged to distinguish small GIST from other gastrointestinal benign tumors [46, 47].

#### Gastrointestinal Schwannoma

Gastrointestinal schwannoma is thought to originate as an over proliferation of Schwann cells within the autonomic nervous system of the gastrointestinal tract and often manifests as a prominent lymphatic cuff-like mass [48]. Gastrointestinal schwannoma are relatively rare, accounted for 4% of all benign gastric tumors, with the most common site being the stomach, followed by the colon and the rectum [49]. Gastrointestinal schwannoma usually presented as a submucosal lesion with an endogenous or exogenous growth pattern, and its common CT presentation is homogeneous and moderate enhancement, which means calcification, cystic changes, hemorrhage, and necrosis are rarely observed in gastrointestinal schwannoma [50, 51].

#### Gastrointestinal leiomyoma

**Fig. 10:**
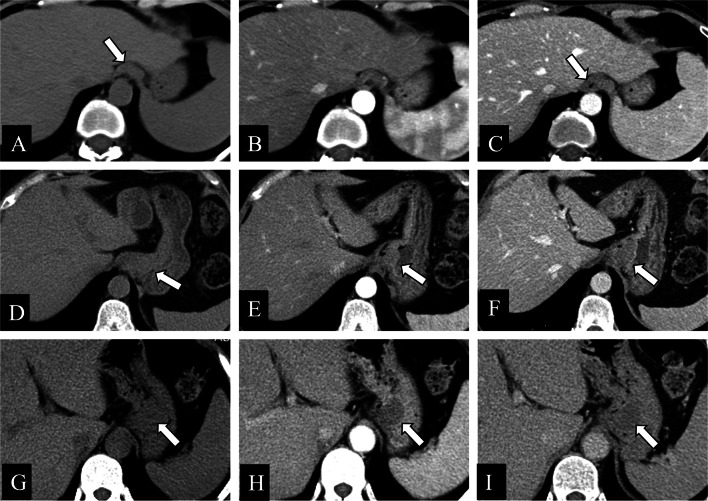
**a–c** Heterotopic pancreas in a 48-year-old female. Axial contrast-enhanced CT images obtained during the plain (**a**) /arterial (**b**) /venous (**c**) phase showed a flat mass (white arrows) in the gastric cardia, with a tendency to grow horizontally. **d–f** Gastrointestinal leiomyoma in a 42-year-old male. Axial contrast-enhanced CT images also demonstrated a same flat mass (white arrows) in the gastric cardia, but with a vertical growth tendency compared to the horizontal axis. **g–i** Gastrointestinal leiomyoma in a 48-year-old female. Axial contrast-enhanced CT images also revealed a vertically growing and round mass (white arrows) in the gastric cardia

Gastrointestinal leiomyoma is benign, not requiring surgery unless obstruction or compression symptoms. The most common location of gastrointestinal leiomyoma is the gastroesophageal junction, while in the stomach it is usually located in the cardia [52]. Gastrointestinal leiomyoma often displays as a small homogeneous hypodense mass with mild to moderate enhancement, often with endoluminal growth pattern (Fig. 10) [53].

#### Gastrointestinal neuroendocrine tumors

Gastrointestinal neuroendocrine tumor is a rare and slow-growing tumor derived from intestinal villous-like cells [54]. More than two-thirds of small bowel neuroendocrine tumors occur in the terminal ileum within 60 cm of the ileocecal valve [55]. Neuroendocrine tumor of the duodenum or jejunum varies biologically and clinically [56]. Carcinoid syndrome generally occurs when jejunal neuroendocrine tumor metastasize to the liver [57]. Appendiceal neuroendocrine tumor is most often encountered after appendectomy [58]. Colonic neuroendocrine tumor is usually large at the time of diagnosis, i.e., tumor with local or distant metastases [59].

Characteristic CT signs help to distinguish GHP from other submucosal lesions in the gastrointestinal tract that are seem similar oval or flat in shape. GHP is mostly a small, solitary lesion located distal to the gastric antrum with a large LD/SD ratio, and its density and enhancement pattern resemble that of the normal pancreas. GIST is mostly a mixed-growing, abundant bloody supply mass in the submucosa of the gastrointestinal tract, commonly with mucosal ulceration and amorphous calcification. Gastrointestinal schwannoma generally shows as a round, homogeneous, hypodense lesion in the stomach. Gastrointestinal leiomyoma customarily presents as a small, hypodense, mild enhancement, and intraluminal-growing lesion in the esophagogastric junction. Neuroendocrine tumor of the gastrointestinal tract varies according to its classification and location and thus imaging performance. Familiarity with their respective imaging manifestations can assist clinicians in making a diagnosis before treatment.

## Conclusions

GHP is a variant congenital of development, which is rarely detected on prospective imaging, while most cases are discovered at surgery or autopsy. Comprehension of the common sites of occurrence and imaging features of GHP is key aspects of accurate preoperative diagnosis. Clinician and radiologist should also be aware of the histoembryological and pathological features of GHP, mastering with clinical manifestations and related complications of GHP, and clear about the diagnostic points of differentiation between GHP and other gastrointestinal submucosal tumors, to avoid as much as possible underdiagnosis and misdiagnosis of such disease during work.

## Data Availability

The data and material are included in this manuscript.

## Abbreviations

CT: Computed tomography
EUS: Endoscopic ultrasonography
GGT: Gastroduodenal glomus tumor
GHP: Gastrointestinal heterotopic pancreas
GIST: Gastrointestinal stromal tumor
GST: Gastric stromal tumors
HP: Heterotopic pancreas
LD: Long diameter
MRCP: Magnetic resonance cholangiopancreatography
MRI: Magnetic resonance imaging
SD: Short diameter
